# Supplementary material for: A Likelihood Approach for Real-Time Calibration of Stochastic Compartmental Epidemic Models
Source: PLoS Comput Biol. 2017 Jan 17;13(1):e1005257. doi: 10.1371/journal.pcbi.1005257 (PMC5240920; doi:10.1371/journal.pcbi.1005257)
Supplement: S1 File — (TAR.GZ) [file pcbi.1005257.s014.tar.gz › HSPH_Online-SI-Revision/Additions/EnKF08/n100-extreme_noisy/n100-extreme_noisy_EnKF_table4.pdf]

1	R0	{2.76052, 16.3913}	{2.6622, 8.67997}	{{1.87766, 7.03969}, {3., 9.71479}}	{14.6659, 47.0453}	inf	11577.	7547.95	{430.728, 31720.7}	98.4627	0.768718	newly	4368.49	6121.42	{300., 28407.}	138.704	0.593505	Reff	0.335762	0.750907	{0.0370112, 1.87672}	150.31	ar	92090.	184655.	{161280., 265947.}	100.516
2	R0	{2.18202, 15.1734}	{2.6559, 5.25295}	{{1.84995, 2.07775}, {3., 7.27796}}	{28.409, 65.3805}	inf	9523.	18008.4	{7483.23, 43817.3}	124.733	0.332731	newly	3796.	21604.4	{5047., 46984.}	478.336	0.614589	Reff	0.491915	0.859393	{0.388663, 1.84994}	85.2971	ar	84264.	222377.	{180041., 280542.}	163.905
3	R0	{2.57891, 12.5971}	{2.48059, 7.17258}	{{2.1038, 3.6266}, {3., 8.28244}}	{20.4671, 43.0617}	inf	6810.	2363.58	{389.788, 3090.8}	76.9917	0.688779	newly	3181.	2094.62	{358., 4075.}	69.3895	0.484989	Reff	0.320791	0.651593	{0.060825, 2.07197}	156.892	ar	90302.	181832.	{162909., 246346.}	101.36
4	R0	{2.5856, 9.28508}	{2.36899, 7.3156}	{{1.87737, 0.104082}, {3., 13.5237}}	{23.4432, 43.9144}	inf	4198.	6517.52	{949.7, 13119.6}	115.348	0.356093	newly	2428.	7603.24	{855., 20103.}	251.679	0.382451	Reff	0.287622	0.661156	{0.123455, 1.87735}	153.472	ar	90312.	197551.	{173295., 267130.}	118.743
5	R0	{2.4406, 12.1341}	{2.25888, 7.06821}	{{0.629186, 5.53002}, {3., 7.76648}}	{36.3257, 41.7492}	inf	8911.	4445.36	{1275.43, 11014.3}	73.241	0.574502	newly	4440.	3540.24	{899., 8031.}	79.7964	0.456123	Reff	0.386079	0.474577	{0.119584, 0.706239}	53.2488	ar	88597.	178019.	{166580., 201794.}	100.931
6	R0	{2.72155, 15.4094}	{2.68568, 4.85917}	{{1.88783, 0.575202}, {3., 19.6134}}	{14.8233, 78.4289}	inf	11194.	6830.59	{831.232, 8976.62}	95.4602	0.646615	newly	4357.	15016.	{866., 89569.}	299.852	0.422146	Reff	0.34629	0.685597	{0.193869, 1.88754}	117.927	ar	91682.	182004.	{161740., 254842.}	98.5163
7	R0	{2.63781, 14.3935}	{2.09755, 6.90876}	{{0.56715, 0.79909}, {3., 13.4909}}	{36.6835, 52.0009}	inf	9306.	13901.7	{1097.06, 54266.2}	115.822	0.389318	newly	3953.	12723.5	{842., 35743.}	244.654	0.409347	Reff	0.327906	0.52339	{0.160499, 0.714474}	88.1485	ar	91192.	183440.	{159881., 269543.}	101.158
8	R0	{2.82391, 16.104}	{2.67473, 4.81027}	{{1.83526, 0.0829442}, {3., 19.644}}	{13.6386, 76.4999}	inf	11648.	3310.62	{1048.91, 5453.87}	83.328	0.755008	newly	4404.	7999.65	{667., 18508.}	162.745	0.44749	Reff	0.322067	0.567533	{0.124064, 1.15748}	114.245	ar	92664.	164648.	{152847., 179616.}	77.6832
9	R0	{2.80645, 14.6457}	{2.60661, 13.567}	{{1.72126, 4.04714}, {3., 19.5406}}	{16.5002, 42.1296}	inf	11375.	4653.73	{758.329, 7428.72}	81.387	0.761779	newly	4649.	2213.05	{310., 3748.}	78.2274	0.687909	Reff	0.325885	0.584553	{0.0220264, 1.68811}	154.631	ar	92567.	172959.	{151860., 225963.}	86.8475
10	R0	{1.86213, 5.4271}	{2.40746, 11.0997}	{{1.48389, 7.41591}, {3., 16.4474}}	{37.5948, 104.523}	inf	1169.	10319.3	{3411.18, 14460.1}	788.065	0.779807	newly	1101.	6229.99	{1242., 10783.}	471.963	0.567467	Reff	0.480541	0.568042	{0.105412, 1.16033}	67.7356	ar	75362.	205443.	{183290., 279776.}	172.608
11	R0	{2.27349, 14.6338}	{2.65856, 7.44687}	{{2.29021, 0.303452}, {3., 17.521}}	{28.1094, 62.0606}	inf	9305.	8200.	{535.169, 16525.9}	100.1	0.573235	newly	3974.	9550.59	{669., 24460.}	203.872	0.515022	Reff	0.44615	0.591164	{0.0970205, 1.19203}	83.9121	ar	85805.	196339.	{173903., 215035.}	128.82
12	R0	{2.77261, 14.1894}	{2.27939, 7.03407}	{{0.516905, 3.58824}, {3., 11.8791}}	{29.2456, 50.4273}	inf	10089.	5822.46	{992.787, 14296.7}	75.4829	0.569864	newly	4154.	5791.22	{964., 11851.}	119.749	0.448387	Reff	0.306207	0.471121	{0.0799769, 0.710436}	92.5415	ar	92508.	173964.	{164314., 199447.}	88.0529
13	R0	{2.65402, 19.3935}	{2.32496, 5.04638}	{{1.38861, 3.39752}, {3., 6.23202}}	{27.5204, 73.979}	inf	15461.	12514.7	{4474.34, 45747.6}	86.092	0.465214	newly	4998.	17003.7	{4093., 58168.}	254.344	0.324367	Reff	0.43088	0.692624	{0.0504176, 1.36749}	108.511	ar	91184.	206668.	{176737., 298182.}	126.65
14	R0	{1.75297, 4.45354}	{2.03836, 7.05486}	{{0.742063, 5.49217}, {3., 7.88331}}	{61.9717, 58.4101}	inf	1073.	3323.36	{745.771, 6917.33}	223.514	0.37804	newly	1153.	2687.13	{487., 5528.}	161.441	0.325527	Reff	0.522297	0.380053	{0.13402, 0.706523}	47.2219	ar	71263.	183722.	{172358., 198330.}	157.808
15	R0	{1.99928, 7.1501}	{2.28086, 7.2816}	{{0.678848, 5.53505}, {3., 7.9668}}	{53.9896, 14.1071}	inf	2594.	7172.94	{1543.15, 23668.6}	195.522	0.295837	newly	1932.	5383.08	{1162., 18045.}	202.959	0.318122	Reff	0.446419	0.391967	{0.0421971, 0.737004}	75.7	ar	79602.	185131.	{167162., 238814.}	132.571
16	R0	{2.67398, 10.3134}	{2.1724, 3.44612}	{{0.511364, 2.9813}, {3., 4.86496}}	{34.8516, 66.586}	inf	4744.	13522.4	{2179.01, 45617.1}	213.999	0.389006	newly	2584.	14712.3	{2479., 50011.}	474.535	0.542939	Reff	0.275794	0.35482	{0.0957167, 0.611223}	54.4036	ar	91289.	199965.	{167409., 281025.}	119.047
17	R0	{2.44634, 16.3226}	{2.41278, 5.33867}	{{1.36376, 3.40508}, {3., 6.49176}}	{30.341, 67.2928}	inf	11754.	9239.31	{2663.31, 6623.29}	92.5051	0.49651	newly	4470.	10021.	{3315., 18127.}	149.27	0.293586	Reff	0.431632	0.686509	{0.112336, 1.25044}	92.0433	ar	88710.	186854.	{162559., 232966.}	110.634
18	R0	{2.02168, 10.1676}	{2.79631, 6.59048}	{{2.13835, 0.743332}, {3., 16.546}}	{38.3162, 71.5346}	inf	6854.	6766.75	{1249.03, 12118.5}	85.2639	0.408325	newly	4020.	18424.9	{671., 85325.}	412.365	0.561717	Reff	0.515609	0.937985	{0.210481, 2.13833}	110.953	ar	80299.	208864.	{178090., 277040.}	160.107
19	R0	{2.89969, 15.009}	{2.66108, 8.89151}	{{1.9087, 0.395173}, {3., 19.4223}}	{12.4491, 65.3869}	inf	11247.	6510.65	{2001.04, 6940.3}	81.8289	0.525541	newly	4512.	11726.8	{829., 31634.}	228.991	0.508157	Reff	0.292405	0.72429	{0.187493, 1.90744}	160.798	ar	93349.	181868.	{160680., 251450.}	94.8263
20	R0	{2.68933, 7.34928}	{2.0868, 7.86052}	{{0.0338399, 3.68121}, {3., 17.6116}}	{37.0257, 46.8093}	inf	2277.	14740.1	{1042.22, 49789.3}	566.127	0.634755	newly	1542.	11474.6	{1002., 38663.}	659.917	0.655758	Reff	0.246316	0.42766	{0.0135409, 0.71822}	141.706	ar	91574.	202678.	{175441., 257429.}	121.327
21	R0	{2.15352, 9.28074}	{2.16169, 7.22007}	{{0.54361, 5.56158}, {3., 8.03741}}	{49.9334, 22.2038}	inf	5169.	5430.04	{1181.29, 14485.5}	88.7048	0.413826	newly	3172.	4200.99	{874., 11857.}	102.098	0.388031	Reff	0.421315	0.409814	{0.084821, 0.756983}	73.4545	ar	83845.	178864.	{162916., 210825.}	113.328
22	R0	{2.21953, 9.04387}	{2.47634, 3.6498}	{{1.06573, 3.26731}, {3., 4.64553}}	{36.0628, 59.6433}	inf	4637.	12817.4	{1530.95, 36175.7}	206.348	0.419651	newly	2827.	15271.7	{1438., 45144.}	458.485	0.612436	Reff	0.397451	0.536307	{0.200427, 0.766093}	56.1084	ar	84856.	184492.	{158954., 259749.}	117.418
23	R0	{2.21987, 14.5347}	{2.55613, 5.26016}	{{1.61264, 2.31075}, {3., 8.5326}}	{32.6466, 63.8096}	inf	9287.	12035.6	{1020.37, 34164.8}	107.867	0.473411	newly	3912.	12927.1	{689., 36501.}	267.092	0.520219	Reff	0.465951	0.625349	{0.155155, 0.85023}	64.9727	ar	85067.	202877.	{167902., 273423.}	138.491
24	R0	{2.84268, 17.9659}	{2.59317, 6.06756}	{{0.955161, 3.32301}, {3., 9.02202}}	{17.6321, 66.2274}	inf	14057.	5215.95	{772.282, 6731.91}	73.9205	0.639514	newly	4888.	5817.32	{1161., 15201.}	80.1956	0.35138	Reff	0.351242	0.70103	{0.254477, 1.33098}	111.978	ar	92836.	169244.	{156787., 190862.}	82.3043
25	R0	{2.52706, 10.3679}	{1.84993, 6.57318}	{{0.419387, 3.62301}, {3., 11.9846}}	{47.3817, 44.3972}	inf	5201.	7044.54	{815.453, 13009.4}	108.475	0.398519	newly	2743.	8695.45	{740., 9270.}	258.376	0.374543	Reff	0.316085	0.504127	{0.0852025, 0.740642}	97.6109	ar	89623.	181657.	{167839., 196632.}	102.69
26	R0	{2.62835, 19.1088}	{2.361, 5.05309}	{{1.47156, 3.37758}, {3., 6.33855}}	{26.5743, 73.5562}	inf	14411.	3286.48	{1234.1, 4244.78}	87.8217	0.865422	newly	4701.	4245.32	{1219., 7050.}	94.2536	0.452766	Reff	0.418354	0.568378	{0.132649, 1.46722}	65.688	ar	90908.	179247.	{162372., 224632.}	97.1738
27	R0	{2.76831, 19.4155}	{2.75529, 3.21076}	{{2.40435, 1.05311}, {3., 5.75252}}	{10.3461, 83.4629}	inf	12467.	2469.71	{77.4986, 1500.28}	96.9531	1.33857	newly	3937.	4151.32	{111., 7745.}	128.687	0.762784	Reff	0.347063	0.636259	{0.256588, 1.10586}	97.4102	ar	92175.	172480.	{159515., 184841.}	87.1221
28	R0	{2.96609, 16.8903}	{2.27348, 3.41859}	{{0.672575, 2.97681}, {3., 4.99259}}	{24.8143, 79.76}	inf	11510.	7007.12	{1685.4, 12943.6}	74.6271	0.535328	newly	4186.	8418.75	{1621., 18612.}	125.748	0.346684	Reff	0.274037	0.431307	{0.105038, 0.640839}	88.3787	ar	94024.	184228.	{166873., 227900.}	95.9376
29	R0	{2.17046, 14.39}	{2.582, 3.46092}	{{1.85145, 2.60414}, {3., 4.42517}}	{28.0524, 75.9492}	inf	10054.	8958.28	{1047.8, 24373.3}	88.4739	0.464876	newly	4398.	11534.9	{1416., 35575.}	181.075	0.347413	Reff	0.504914	0.456316	{0.111484, 0.908844}	58.3793	ar	84001.	184892.	{159599., 257584.}	120.106
30	R0	{2.88772, 12.4647}	{2.41773, 5.08086}	{{1.83546, 1.1668}, {3., 15.1658}}	{20.1637, 70.0663}	inf	8175.	7803.48	{1315.73, 7868.08}	87.0193	0.40834	newly	3775.	9843.63	{1862., 13220.}	194.94	0.357193	Reff	0.263591	0.567703	{0.141851, 1.61009}	139.141	ar	93273.	193621.	{170569., 252241.}	107.585
31	R0	{2.46831, 8.14308}	{1.98288, 9.99923}	{{0.584225, 3.73517}, {3., 19.0439}}	{43.7919, 53.3146}	inf	3768.	4586.25	{1539.61, 5824.67}	63.9648	0.202054	newly	2494.	3165.99	{469., 3314.}	71.9531	0.245226	Reff	0.317474	0.315124	{0.0501196, 0.522974}	56.1042	ar	88762.	175084.	{167550., 173396.}	97.2512
32	R0	{1.93236, 7.38987}	{2.28593, 5.41981}	{{1.24496, 2.40277}, {3., 6.49132}}	{40.2685, 26.6589}	inf	3204.	6778.22	{539.179, 17261.2}	180.112	0.469862	newly	2353.	7496.27	{511., 16096.}	275.742	0.485352	Reff	0.482588	0.474602	{0.108731, 0.847738}	60.3246	ar	77654.	188293.	{165280., 243916.}	142.477
33	R0	{2.82251, 17.6954}	{2.6342, 9.85278}	{{2.19938, 5.55609}, {3., 15.0304}}	{15.6012, 44.3201}	inf	15003.	2137.4	{644.088, 1519.27}	95.0126	1.21236	newly	5209.	1254.64	{286., 934.}	92.9007	0.958437	Reff	0.365938	0.49461	{0.103335, 0.909206}	76.4212	ar	92745.	162995.	{154688., 180340.}	75.7457
34	R0	{1.87083, 12.3994}	{2.51866, 3.08803}	{{1.75342, 1.70702}, {3., 4.77983}}	{38.7104, 75.0953}	inf	8229.	11042.2	{588.672, 45794.9}	112.202	0.463771	newly	4286.	16144.6	{955., 45466.}	301.877	0.518583	Reff	0.630488	0.778679	{0.20825, 1.68144}	59.656	ar	76282.	203172.	{157543., 282906.}	166.344
35	R0	{2.95779, 16.3003}	{2.61935, 7.26423}	{{2.15411, 3.51124}, {3., 8.86081}}	{13.0121, 55.435}	inf	14081.	4673.87	{438.716, 11856.3}	83.3319	0.883243	newly	5272.	4391.81	{297., 14524.}	100.677	0.67627	Reff	0.314472	0.553283	{0.0400849, 2.15409}	135.248	ar	93873.	181318.	{161300., 256734.}	93.1525
36	R0	{2.18718, 14.261}	{2.41702, 7.32457}	{{0.834709, 3.46061}, {3., 12.6243}}	{43.7494, 48.6391}	inf	9571.	5365.17	{1248.18, 9069.9}	68.5275	0.493149	newly	4189.	5258.82	{818., 10495.}	93.7756	0.383309	Reff	0.488835	0.614605	{0.218257, 0.835656}	53.1859	ar	84379.	188148.	{175585., 211301.}	122.979
37	R0	{2.44912, 9.31118}	{2.53246, 6.4087}	{{2.03838, 0.330612}, {3., 18.0771}}	{21.3392, 78.2333}	inf	4364.	8043.3	{1093.63, 10513.4}	112.934	0.298916	newly	2540.	13739.8	{890., 41028.}	469.933	0.560768	Reff	0.332493	0.553957	{0.0242888, 1.29288}	107.231	ar	88442.	199605.	{177950., 247276.}	125.69
38	R0	{2.05949, 5.72514}	{2.60939, 6.64616}	{{2.03335, 0.26901}, {3., 18.2688}}	{27.564, 111.702}	inf	1447.	10567.1	{2673.91, 13053.1}	636.149	0.684686	newly	1229.	16252.2	{1930., 41548.}	1224.94	0.798557	Reff	0.397749	0.59823	{0.286502, 1.26278}	71.3483	ar	81587.	214155.	{191401., 276832.}	162.487
39	R0	{1.75505, 8.52404}	{2.62765, 6.67008}	{{2.04526, 0.746646}, {3., 17.4585}}	{49.7194, 82.4953}	inf	4589.	7251.67	{569.624, 16810.8}	130.661	0.462703	newly	3152.	11111.2	{636., 23056.}	308.961	0.623858	Reff	0.605615	0.714982	{0.0520221, 2.04524}	87.8166	ar	70925.	186180.	{158979., 252743.}	162.503
40	R0	{2.24724, 13.7861}	{2.4094, 3.30652}	{{1.12339, 3.12901}, {3., 3.53842}}	{35.0288, 76.0155}	inf	8230.	2800.11	{459.077, 5679.92}	82.4892	0.793531	newly	3630.	3796.04	{527., 7747.}	104.918	0.498467	Reff	0.451088	0.429516	{0.150064, 0.710636}	54.2692	ar	85257.	172998.	{160994., 193479.}	102.914
41	R0	{1.77635, 8.62406}	{2.60466, 7.13501}	{{1.87065, 0.690846}, {3., 19.8259}}	{46.6299, 101.233}	inf	4190.	2349.88	{729.421, 3639.05}	56.4182	0.398186	newly	2830.	10004.2	{473., 34009.}	327.854	0.499194	Reff	0.557952	0.610205	{0.077819, 1.85982}	67.7353	ar	72941.	168244.	{150991., 230506.}	130.658
42	R0	{1.80397, 5.4932}	{2.4016, 10.4362}	{{1.60467, 8.60967}, {3., 15.3387}}	{41.8052, 89.9833}	inf	1509.	8773.65	{725.461, 7798.06}	503.998	0.635807	newly	1451.	5536.92	{314., 6556.}	318.178	0.548476	Reff	0.511444	0.468954	{0.096439, 0.545676}	49.9668	ar	73232.	182771.	{161070., 241329.}	149.579
43	R0	{2.83096, 16.7077}	{2.66436, 5.31891}	{{1.96493, 3.21943}, {3., 6.56661}}	{13.9845, 68.1649}	inf	13176.	18674.4	{9833.06, 53927.6}	84.3668	0.242816	newly	4750.	17021.8	{6883., 40528.}	260.263	0.440064	Reff	0.342093	0.399804	{0.0831338, 0.548557}	66.9856	ar	92740.	213136.	{183205., 279607.}	129.821
44	R0	{2.73678, 11.6021}	{2.28341, 11.0128}	{{1.41278, 5.45382}, {3., 14.3939}}	{25.4144, 22.4734}	inf	5400.	9241.81	{1038.14, 25335.5}	113.432	0.350985	newly	2650.	6297.67	{677., 16209.}	177.127	0.39439	Reff	0.263771	0.651625	{0.290951, 1.40895}	152.15	ar	92003.	206841.	{181769., 276480.}	124.819
45	R0	{2.14235, 6.98601}	{1.864, 3.65401}	{{0.625728, 2.83625}, {3., 5.46619}}	{53.8266, 47.7903}	inf	1905.	10443.3	{663.606, 28413.5}	467.027	0.626607	newly	1420.	10559.8	{649., 25826.}	659.799	0.741671	Reff	0.389458	0.298197	{0.0986707, 0.555269}	47.6629	ar	82974.	205995.	{180568., 241549.}	148.265
46	R0	{2.23409, 9.21289}	{2.08263, 7.20386}	{{0.652754, 3.87828}, {3., 8.54677}}	{47.9189, 21.8067}	inf	5803.	4304.32	{310.674, 11342.9}	86.4094	0.573901	newly	3539.	3184.9	{276., 8174.}	89.7824	0.507418	Reff	0.407766	0.44976	{0.10118, 0.713596}	60.0038	ar	85163.	178905.	{167378., 204841.}	110.074
47	R0	{2.12258, 8.15265}	{2.48093, 10.0418}	{{2.0529, 6.85867}, {3., 17.7775}}	{21.9742, 42.9385}	inf	3887.	10073.5	{1695.2, 16411.7}	180.79	0.36589	newly	2681.	6982.78	{1056., 14701.}	191.113	0.388375	Reff	0.420441	0.567893	{0.161816, 1.27697}	73.0206	ar	82690.	194786.	{168968., 264587.}	135.562
48	R0	{2.71771, 10.4636}	{2.56379, 7.33641}	{{1.86023, 0.580778}, {3., 16.837}}	{17.7124, 72.4354}	inf	6665.	11974.4	{1574.25, 15248.7}	118.944	0.331622	newly	3585.	21493.1	{926., 80026.}	532.777	0.593574	Reff	0.28718	0.799878	{0.419855, 1.54987}	182.317	ar	91716.	209503.	{179958., 263857.}	128.426
49	R0	{2.44369, 6.66477}	{1.71602, 8.80439}	{{0.256529, 3.70858}, {3., 19.0973}}	{53.9087, 57.8296}	inf	1262.	4291.	{917.945, 4536.}	253.523	0.391191	newly	921.	3495.91	{578., 4126.}	297.163	0.39963	Reff	0.285399	0.279222	{0.025226, 0.617876}	71.6944	ar	88827.	179738.	{171241., 179799.}	102.346
50	R0	{2.77383, 17.9775}	{2.54634, 3.82463}	{{2.05487, 2.52461}, {3., 4.01129}}	{16.6813, 78.7255}	inf	11601.	8375.21	{1100.65, 24771.8}	92.1624	0.616776	newly	3992.	15743.	{1261., 50937.}	335.401	0.452828	Reff	0.332083	0.78282	{0.138765, 2.05485}	185.29	ar	92334.	198443.	{162625., 258256.}	114.918
